# An integrated case–based leptospirosis surveillance dataset with environmental context from a tropical coastal city in Indonesia, 2018—2023

**DOI:** 10.1016/j.dib.2026.112930

**Published:** 2026-06-06

**Authors:** Kiki Adhinugraha, Vincentia Rizke Ciptaningtyas, Rebriarina Hapsari, Gede Agung Ary Wisudiawan, Wiwik Dwi Lestari, Jeremia B.M.P. Hutapea, Syefi Berliana Pakpahan, David Taniar

**Affiliations:** a Department of Computer Science and Information Technology, La Trobe University, Australia; b Department of Clinical Microbiology, Faculty of Medicine, Universitas Diponegoro, Indonesia; c Diponegoro National Hospital, Indonesia; d KRMT Wongsonegoro Hospital, Indonesia; e Faculty of Informatics, Telkom University, Indonesia; f Semarang City Health Office, Indonesia; g Faculty of Information Technology, Monash University, Australia; h Victorian Heart Institute (VHI), Australia

**Keywords:** Zoonotic disease, Public health monitoring, Urban flooding, Rodent-borne infection, Climate variability, Case investigation, Tropical health, Disease transmission

## Abstract

This dataset contains anonymised leptospirosis surveillance records collected in Semarang, Central Java, Indonesia between 2018 and 2023. Case data were derived from routine public health surveillance conducted by community health centre teams. The primary dataset includes patient demographic characteristics, case classification status, documented clinical events expressed as day offsets relative to registration date, exposure history to standing and contaminated water, animal contact variables, personal protective equipment usage, bathing practices, wound conditions, and household environmental characteristics.

Spatial context is provided at the kelurahan level, the smallest formal government administrative unit in Indonesia with defined spatial boundaries, enabling geospatial analysis while preserving individual privacy. Secondary contextual datasets include administrative boundaries, land use classification, healthcare facility locations, flood related indicators, population statistics, and monthly weather variables such as temperature, relative humidity, and rainfall.

Personally identifiable information and direct healthcare facility identifiers were removed prior to dataset construction. This dataset supports research in disease surveillance, spatial epidemiology, environmental health, zoonotic transmission, and climate sensitive disease modelling in tropical urban settings.

Specifications Table

**Table:** 

Subject	Health Sciences, Medical Sciences & Pharmacology
Specific subject area	Public health surveillance and spatial epidemiology of zoonotic disease in urban tropical settings.
Type of data	Table, Cleaned, Anonymised
Data collection	Leptospirosis case data were collected through routine surveillance by community health centre teams. After case registration, surveillance officers visited the patient’s residence to assess environmental conditions and conduct structured interviews with patients and close contacts. Follow up observations and reported clinical changes were recorded over time. Data include pre registration exposure history and post registration follow up observations. Personally identifiable information was removed during dataset construction.
Data source location	Case Data: Leptospirosis case records were collected from routine surveillance conducted by community health centres in Semarang, Central Java, Indonesia. The study area is located between 110° 16′ 10.96″ E to 110° 30′ 18.81″ E and 7° 6′ 55.67″ S to 6° 56′ 1.47″ S. Administrative Boundaries, Elevation and Land Use: Administrative boundary, Elevation, and land use spatial data were obtained from Geospasial Untuk Negeri portal (https://tanahair.indonesia.go.id/portal-web/unduh). Healthcare Facilities: Healthcare facility location data were collected from the Semarang City Health Office in 2025. Flood Risk Data: Flood hazard and risk related variables were obtained from the Regional Disaster Management Agency, Badan Penanggulangan Bencana Daerah (BPBD) Semarang in 2022. Population Data: Kecamatan level population data were obtained from Semarang City Statistic Agency, Badan Pusat Statistik (BPS) Semarang. Weather Data: Monthly weather data including relative humidity, temperature, and rainfall were obtained from Semarang City Meteorological Agency, Badan Meteorologi, Klimatologi, dan Geofisika (BMKG) Semarang.
Data accessibility	Repository name: A georeferenced leptospirosis case dataset with environmental and exposure attributes in Semarang, Indonesia, 2018 to 2023 Direct URL to data:https://doi.org/10.6084/m9.figshare.31386364
Related research article	No related articles.

## 1 Value of the Data

• These data provide a structured, individual level surveillance dataset of confirmed, probable, and suspected leptospirosis cases linked to environmental, behavioural, and temporal variables. The integration of case attributes, documented symptom timing, exposure indicators, and household environment variables enables detailed epidemiological analysis beyond routine case counts.
• The dataset supports spatial analysis at the kelurahan level, the smallest official administrative unit in Indonesia, allowing researchers to link cases with land use, population density, flood risk, healthcare access, and meteorological conditions. This structure facilitates geospatial modelling while preserving patient privacy.
• The inclusion of exposure flags for standing water, dirty water, animal contact, and personal protective equipment enables behavioural risk assessment and comparative studies across regions. Researchers can reuse these variables to examine transmission pathways in urban tropical environments.
• Temporal offsets from the registration date allow reconstruction of documented symptom changes and healthcare seeking timelines. This enables exploratory analyses of documented symptom timing, disease course, delay patterns, and surveillance responsiveness.
• The harmonised secondary datasets, including administrative boundaries, land use classification, healthcare facilities, weather, and population data, provide a reproducible framework for integrated public health analysis and for comparative studies in other endemic settings.

## 2 Background

Leptospirosis is a zoonotic bacterial disease prevalent in tropical and subtropical regions and remains an important public health concern. Transmission is associated with environmental exposure, flooding, and interactions among human, animal, and environmental reservoirs [1]. Several datasets support leptospirosis research, including clinical surveillance records, demographic and exposure data, pathogen genomic sequences, and spatial analyses of disease distribution [[2], [3], [4]]. These resources contribute to understanding disease epidemiology and transmission in different settings. However, integrated datasets linking case-based surveillance records with environmental and contextual variables remain limited.

Leptospirosis remains a recurrent public health issue in Central Java, Indonesia. The 2023 Executive Health Report of the Central Java Health Office reported incidence rates of 0.76–2.35 per 100,000 population during 2019–2023, with case fatality rates ranging from 11.61% to 16.60% [5]. Semarang, the capital city of Central Java, is a coastal urban area prone to flooding and heavy rainfall, conditions that may influence leptospirosis transmission. Integrating surveillance records from this setting with environmental and contextual variables can enrich existing datasets and support epidemiological and spatial analyses.

The dataset presented here organises case-based surveillance records collected by public health teams and integrates administrative, environmental, population, and meteorological context to support epidemiological and spatial analysis.

## 3 Data Description

The repository contains primary case data, harmonised secondary geospatial and environmental datasets, and supporting documentation. The spatial attributes follow the Indonesian administrative hierarchy: Province → City/Municipality → Subdistrict → Urban Village (Kelurahan). Case records are georeferenced at the Urban Village level, which represents the lowest administrative unit used in this dataset. Repository files are organised into primary data, secondary geospatial data, secondary tabular data, and supporting documentation folders to improve accessibility and reproducibility.

1. Primary Data.
  The file leptospirosis_surveillance_cases_semarang_2018_2023.csv contains 182 de-identified leptospirosis surveillance records and 107 variables from Semarang between 2018 and 2023. Cases were distributed across 83 kelurahan within 16 kecamatan. The surveillance records contain suspect, probable, and confirmed diagnostic stages recorded through routine public health surveillance systems, although explicit confirmed diagnostic timing was available for only one released record. This reflects real-world surveillance practice in endemic tropical settings, where laboratory confirmation may be constrained by diagnostic availability, timing of presentation, referral patterns, and resource limitations. The file includes demographic attributes, occupation group, documented clinical events expressed as day offsets relative to the original registration date, first documented symptom indicators, structured exposure variables, bathing and wound information, and household environmental indicators. Missing values were retained where information was unavailable, not recorded, or could not be harmonised reliably. The variable structure and coding conventions are documented in detail in metadata.txt and the accompanying CSV and markdown data dictionary files.
2. Secondary Geospatial Data.

a. The semarang_administrative_boundaries_kelurahan.geojson file contains polygon boundaries of kelurahan within Semarang. Each feature includes hierarchical administrative identifiers (province, city, kecamatan, kelurahan), environmental attributes such as area (sqkm), shoreline flag, altitude summaries, assigned primary puskesmas name, distance to puskesmas, and flood related indices. Geometry is provided in WGS84 coordinate system.
b. The semarang_landuse_polygons.geojson file contains land use polygons nested within kelurahan boundaries. Each feature contains administrative identifiers, polygon area (sqkm), a harmonised English land use category, and a more specific land use purpose. Geometry is provided in WGS84.
c. The semarang_healthcare_facilities.geojson file contains point locations of healthcare facilities in Semarang, including Puskesmas and hospitals. Attributes include facility identifier, name, facility type, facility class, and administrative labels. Geometries are provided in WGS84. Facilities are not directly linked to individual case records.

3. Secondary Tabular Data.

a. The semarang_population_estimates_2018_2023.csv file contains annual population counts at kecamatan level from 2018 to 2023, with kelurahan level estimates derived using residential land area proportional allocation. Values represent analytical estimates rather than official census counts.
b. The semarang_monthly_weather_2018_2023.csv file contains monthly city level meteorological summaries from 2018 to 2023, including relative humidity (%), temperature ( °C), and rainfall (mm). Data apply to the entire city rather than individual kelurahan.

4. Supporting Documentation.

a. The leptospirosis_case_investigation_form.pdf file is the original standardised case investigation form used by puskesmas staff for field data collection.
b. The metadata.txt contains comprehensive documentation describing all files, variables, coding rules, administrative hierarchy, de-identification measures, and conventions.
c. The data_dictionary_leptospirosis_semarang_2018_2023.csv provides structured variable-level documentation including data types, linkage roles, temporal offset interpretation, NULL handling rules, harmonisation notes, derivation procedures, and analytical cautions for all released datasets.
d. The data_dictionary_leptospirosis_semarang_2018_2023.md provides a human-readable narrative version of the data dictionary, including dataset summaries, coding conventions, temporal interpretation rules, descriptive statistics, and contextual explanations to support reproducibility and secondary data reuse.

**Fig. 1:**
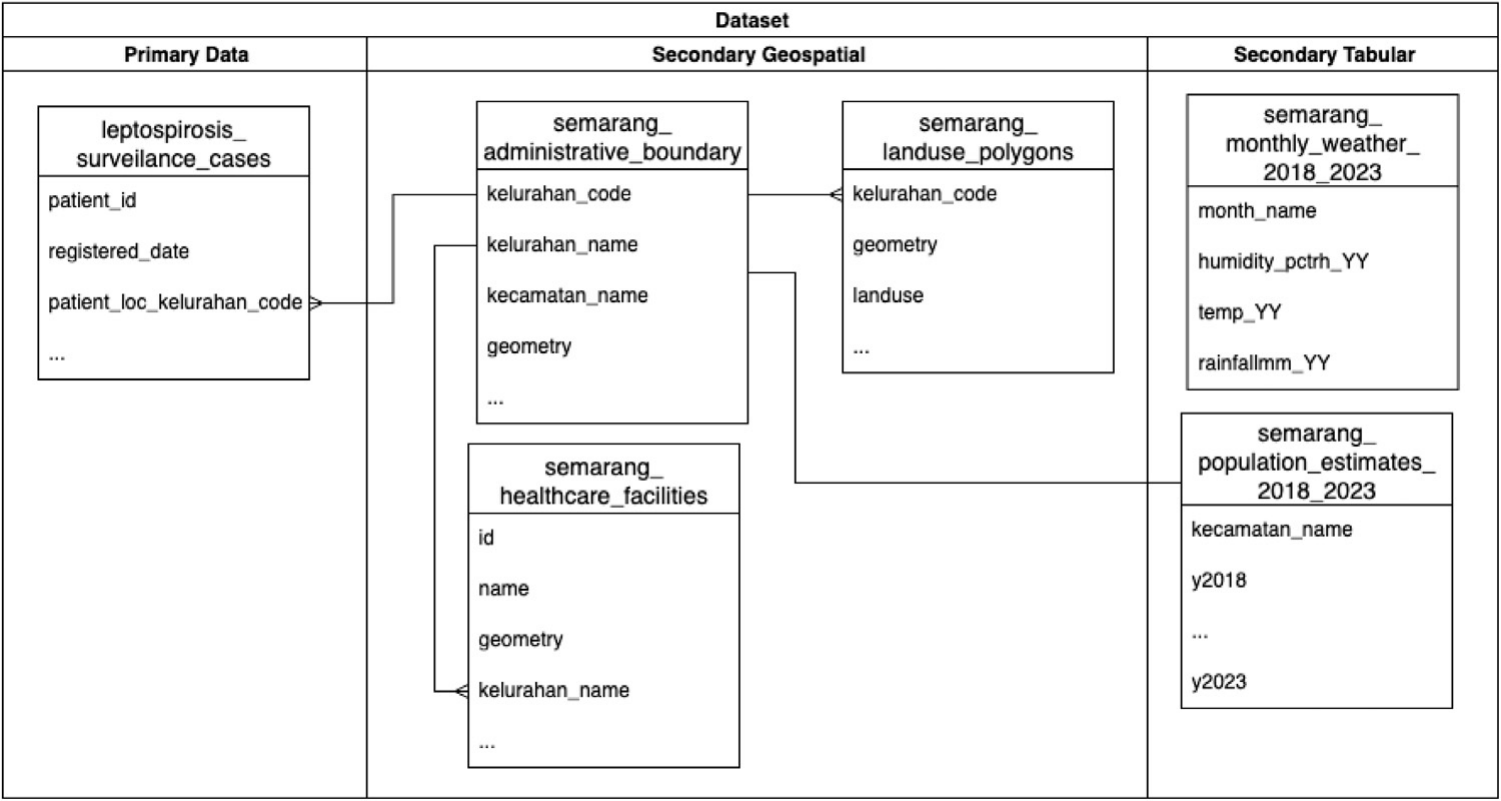
Data model illustrating relationships among primary case data and secondary contextual datasets.

The primary linkage is between cases.patient_loc_kelurahan_code and administrative.kelurahan_code, which anchors each case to a kelurahan boundary. Land use polygons are linked through landuse.kelurahan_code to the administrative layer. Healthcare facilities are associated with administrative areas using kelurahan_name. Population data are linked at kecamatan level using kecamatan_name from the administrative layer. The weather dataset is provided as city level contextual data and is not directly linked to individual administrative units. An interactive visualisation of the surveillance data is also available using Shinyapps at https://geohub.shinyapps.io/Semarang_Leptospirosis_Data/ Fig. 1.

## 4 Experimental Design, Materials and Methods

The dataset was compiled through a structured workflow consisting of primary case data preparation and secondary contextual data integration. Primary data were derived from routine leptospirosis surveillance records collected by Puskesmas staff (community-based primary healthcare centres operating at the sub-district level) in Semarang between 2018 and 2023. Case records are presented at the kelurahan level, an officially recognised urban village administrative unit nested within a kecamatan (sub-district). Secondary datasets were obtained from official administrative, environmental, demographic, and meteorological sources and were processed to ensure structural compatibility with the primary case table. All datasets were harmonised, standardised, and exported to analysis-ready formats in accordance with defined coding rules and de-identification procedures.

1. Primary data acquisition and preparation.

1.1 Surveillance data collection.
  Case records were generated through routine leptospirosis surveillance conducted by Puskesmas teams in Semarang. Upon registration of a suspected case, surveillance staff conducted residential visits and structured interviews to document patient demographic profile, recent activities reported during the surveillance investigation period, potential animal or human contact, environmental exposure, and household conditions.
  The investigation also included follow up monitoring after registration, during which documented symptom changes, laboratory confirmation status, hospitalisation, and final outcome were recorded. Data were captured using a standardised investigation form used across Puskesmas units.
1.2 Data entry and source format.
  At the facility level, records were entered manually in spreadsheet files. In most Puskesmas, one file corresponded to one patient. A smaller number of facilities used a dedicated local application to record surveillance information. The majority of records followed a common template structure.
  All original entries were recorded in Bahasa Indonesia. Some notes contained local dialect expressions or non standard abbreviations.
1.3 Data consolidation and harmonisation.
  Individual patient files were consolidated into a single structured dataset. During consolidation, the following procedures were applied:

• Translation of non English and mixed language entries into standardised medical terminology.
• Harmonisation of categorical variables using controlled vocabulary.
• Grouping of raw occupation descriptions into broader analytical categories to improve privacy protection and analytical consistency.
• Conversion of qualitative responses such as “ya”, “tidak”, and “tidak tahu” into structured binary or missing values.
• Standardisation of exposure descriptions into structured indicator fields and short descriptive notes.
• Manual review of potentially inconsistent temporal or categorical entries through cross checking of structured fields and available narrative notes by multiple reviewers.
• Retention of unresolved ambiguous values as missing rather than applying unsupported corrections.

Free text narratives were retained only where necessary for contextual meaning. Personal identifiers contained within narrative text were removed.

1.4 Temporal transformation.
  The original registration date was used as the temporal baseline for calculating all day offset variables prior to de-identification. After offset calculation, the released registered_date field was reduced to month and year only. Records containing incomplete or internally inconsistent dates were manually reviewed and, where possible, harmonised through cross-validation by multiple reviewers using structured surveillance fields and supporting notes. This transformation preserves temporal ordering while reducing calendar precision to minimise re-identification risk.
1.5 Privacy protection measures.
  The following steps were implemented prior to dataset release:

• Removal of patient names and direct identifiers.
• Replacement of full date of birth with age at registration.
• Removal of detailed address information and retention of kelurahan level location only.
• Removal of healthcare facility identifiers associated with individual cases.
• Removal of narrative fields containing personal identifiers.

2. Secondary data acquisitions and preparation.

2.1 Administrative boundary data.
  Kelurahan boundary data were obtained in vector format [6]. The dataset was reviewed and cleaned prior to integration.
  Administrative names were cross validated against the case dataset because minor spelling variations and phonetic differences were observed between surveillance records and boundary files. Standardised naming was enforced to ensure consistent linkage through kelurahan_code.
  Non essential attributes from the original boundary file were removed. Additional contextual variables were incorporated:

• Coastal exposure flag, derived by identifying kelurahan polygons intersecting the coastline.
• Elevation metrics, derived by overlaying kelurahan polygons with elevation raster data and computing mean and majority altitude values.
• Flood hazard, vulnerability, capacity, and risk indicators, derived from official flood-risk maps provided by BPBD Semarang in colour-coded PDF/raster format. The original maps did not provide complete numeric values for every kelurahan. Therefore, flood-related values were interpreted from mapped colour classes and estimated using the proportional area coverage of each kelurahan intersecting the mapped flood categories. These variables should be interpreted as contextual spatial estimates rather than direct hydrological measurements.

All spatial processing was conducted in a GIS environment and exported in WGS84 coordinate system.

2.2 Land use data.
  Land use polygons were obtained in vector format and processed separately from the administrative boundary layer.
  Land use categories were translated into standardised English terminology. Where necessary, detailed categories were grouped into harmonised classes for analytical consistency. An additional purpose column was added to preserve more specific land use information.
  Land use polygons were spatially intersected with kelurahan boundaries to ensure that each polygon record corresponds to a specific kelurahan. This allows fine granularity land use analysis within each administrative unit.
  Polygon areas were calculated in square kilometres and stored in the attribute table.
2.3 Healthcare facility data.
  The healthcare facility list was provided by the local health authority, whereas geographic coordinates were obtained manually via geocoding in Google Maps.
  Each facility was represented as a point geometry in WGS84 coordinate system. Facility type and classification were standardised according to the Indonesian healthcare system, distinguishing Puskesmas and hospitals, and recording the official facility class.
  Facilities were included as contextual data and are not directly linked to individual patient records.
2.4 Population data.
  Annual population data were obtained at kecamatan level. The dataset was restructured into a pivot format where each year is presented as a separate column.
  Kelurahan level population estimates were derived using proportional allocation based on residential land area within each kecamatan. Residential land area was calculated from the processed land use layer. The resulting values represent analytical estimates and should not be interpreted as official census counts at kelurahan level.
2.5 Weather data.
  Monthly city level weather data were obtained from the national meteorological authority. Variables include relative humidity, average temperature in degrees Celsius, and total rainfall in millimetres.
  The dataset was reshaped into a pivot structure where each year specific measurement is represented in a separate column. Weather data remain at city level and are not spatially disaggregated. Accordingly, these variables should not be interpreted as localised kelurahan-specific weather observations.

## Limitations

Several limitations should be considered when using this dataset. The surveillance records represent suspect, probable, and confirmed cases of leptospirosis reported through routine public health surveillance systems. As with many infectious disease surveillance datasets, reported cases may not capture the full number of infections occurring in the population due to variations in healthcare-seeking behaviour, diagnostic availability, and reporting practices.

Primary surveillance records were originally documented in free text format, including typographical variations, mixed language expressions, and incomplete entries. During data preparation, interpretation and harmonisation were required to standardise variables into structured fields. Although cross-validation and multi-reviewer checks were performed, minor misclassification cannot be completely excluded, and missing values remain in some variables to preserve the original reporting structure.

Case records are georeferenced at the urban village administrative level to protect privacy. Environmental and flood risk indicators were derived from secondary datasets and digitised map sources, which may introduce minor approximations during spatial integration.

Finally, the dataset represents surveillance data from a single metropolitan region in Indonesia, and findings derived from analyses should be interpreted within the context of the local setting.

## Ethics Statement

This study involved human subjects and was conducted in accordance with the principles of the Declaration of Helsinki. Ethical approval was obtained from the Institutional Ethics Committee of Diponegoro University, Indonesia (Approval No:149/EC/KEPK/FK-UNDIP/VI/2025). The 2025 ethics approval specifically covered retrospective consolidation, harmonisation, de-identification, and public release of routine surveillance records collected between 2018 and 2023.

Data were collected as part of routine public health surveillance. Written informed consent was obtained from participants by the local community-based healthcare surveillance team at the time of the investigation. For this data publication, all records were fully de-identified prior to consolidation and release. Direct personal identifiers were removed, and temporal and spatial precision were reduced to minimise re-identification risk.

## CRediT Author Statement

**KA, VRC, RH, DT:** Conceptualization. **KA, JBMPH, SBP:** Software. **KA, GAAW, VRC, RH:** Validation. **GAAW, WDL, JBMPH, SBP:** Resources. **KA, GAAW, JBMPH, SBP:** Data Curation. **KA, VRC, RH, DT:** Writing – Review & Editing.

## Declaration of generative ai and ai-assisted technologies in the manuscript preparation process

During the preparation of this work the author(s) used ChatGPT(OpenAI) in order to improve language clarity and readability. After using this tool/service, the author(s) reviewed and edited the content as needed and take(s) full responsibility for the content of the published article.

## Data Availability

FigshareAn integrated case–based leptospirosis surveillance dataset with environmental context from a tropical coastal city in Indonesia, 2018—2023 (Original data)

## References

[1] Griebsch C., Norris J., Ward M.P (2025). Emerging human and canine leptospirosis in New South Wales: insights from a one health geospatial study. Sci. One Health.

[2] Agampodi S., Warnasekara J., Siribaddana S., Kularatna S., Gamage C., Jayasundara D. (2022). Demographic, exposure, clinical, biochemical and diagnostic data of febrile patients recruited for the largest field study on leptospirosis in Sri Lanka. Data Br..

[3] Philip N., Jani J., Azhari N.N., Sekawi Z., Neela V.K (2021). Genomic data of Leptospira interrogans HP358 isolated from rodent captured from the human leptospirosis suspected areas of Selangor state, Malaysia. Data Br..

[4] Esteves S.B., de Oliveira L.M., Guilloux A.G.A., Cortez A., de Masi E., Ferreira I.M.R. (2025). Into the spotlight: a spatial study of potentially underreported leptospirosis among dengue-negative patients in S˜ao Paulo city, Brazil. PLoS Negl. Trop. Dis..

[5] Dinas Kesehatan Provinsi Jawa Tengah. (2023). Health Profile Handbook of Central Java Province 2023 [Buku Saku Kesehatan Provinsi Jawa Tengah 2023]. Retrieved [23 February 2026] from https://dinkesjatengprov.go.id/v2018/dokumen/Buku_Saku_Kesehatan_2023/mobile/index.html.

[6] Badan Informasi Geospasial. (2025). Portal Unduh Data Geospasial Indonesia (Tanah Air Portal). Retrieved [20 August 2025] from https://tanahair.indonesia.go.id/portal-web/unduh.

